# Numerical simulations of targeted delivery of magnetic drug aerosols in the human upper and central respiratory system: a validation study

**DOI:** 10.1098/rsos.170873

**Published:** 2017-12-06

**Authors:** Saša Kenjereš, Jimmy Leroy Tjin

**Affiliations:** Transport Phenomena Section, Department of Chemical Engineering, Faculty of Applied Sciences, and J. M. Burgerscentrum for Fluid Mechanics, Delft University of Technology, Van der Maasweg 9, 2629 HZ Delft, The Netherlands

**Keywords:** computational fluid dynamics, respiratory diseases, human upper and central airways, aerosols, lungs, magnetic drug targeting

## Abstract

In the present study, we investigate the concept of the targeted delivery of pharmaceutical drug aerosols in an anatomically realistic geometry of the human upper and central respiratory system. The geometry considered extends from the mouth inlet to the eighth generation of the bronchial bifurcations and is identical to the phantom model used in the experimental studies of Banko *et al.* (2015 *Exp. Fluids* **56**, 1–12 (doi:10.1007/s00348-015-1966-y)). In our computer simulations, we combine the transitional Reynolds-averaged Navier–Stokes (RANS) and the wall-resolved large eddy simulation (LES) methods for the air phase with the Lagrangian approach for the particulate (aerosol) phase. We validated simulations against recently obtained magnetic resonance velocimetry measurements of Banko *et al.* (2015 *Exp. Fluids* **56**, 1–12. (doi:10.1007/s00348-015-1966-y)) that provide a full three-dimensional mean velocity field for steady inspiratory conditions. Both approaches produced good agreement with experiments, and the transitional RANS approach is selected for the multiphase simulations of aerosols transport, because of significantly lower computational costs. The local and total deposition efficiency are calculated for different classes of pharmaceutical particles (in the 0.1 μm≤*d*_p_≤10 μm range) without and with a paramagnetic core (the shell–core particles). For the latter, an external magnetic field is imposed. The source of the imposed magnetic field was placed in the proximity of the first bronchial bifurcation. We demonstrated that both total and local depositions of aerosols at targeted locations can be significantly increased by an applied magnetization force. This finding confirms the possible potential for further advancement of the magnetic drug targeting technique for more efficient treatments for respiratory diseases.

## 1. Introduction

A rapid worldwide urbanization and the current development of mega-cities in Asia is associated with a significant deterioration of the air quality due to a fast increase of industrial and traffic pollution [1–3]. Especially alarming are the results of numerous studies directly connecting exposure to the long-term ambient air pollution, with a sudden increase in various respiratory diseases among children [4,5]. Inhalation of medical drug aerosols is applied for treating some of the most common respiratory diseases including asthma and chronic obstructive pulmonary disease (according to a World Health Organization fact sheet published in 2016, more than 235 million people have asthma and more than 64 million have chronic obstructive pulmonary disease). Similarly, due to numerous limitations associated with conventional treatments of lung cancer (which is one of the leading cancer killers in both men and women), alternative treatments based on the pulmonary route of drug administration directly to the lungs have recently been proposed [6–8].

Computer simulation-based studies can be a powerful tool in portraying airflow patterns and spatial distributions for different classes of inhaled particles within the human airways. Furthermore, such studies can improve different strategies to deliver inhaled medical drug aerosols to specific predefined locations within the human respiratory system to fight respiratory diseases [9–12]. Ultimately, such simulations can be applied to patient-specific conditions, providing the best strategies to achieve the most efficient delivery of the medical drug aerosols to diseased sites within the upper and central respiratory system. To achieve this goal, we would like to focus on the subject- and patient-specific human airway geometries.

A realistic extra-thoracic airway model was studied in [13]. The single representative geometry was selected from CT scans of five subjects. The final geometry included mouth cavity, larynx and trachea, without bronchial bifurcations. Different inhalation rates of 15, 30 and $60\;l\;\min^{-1}$ were considered with particle diameters ranging between 2 and 20 μm. The fluid flow and particle deposition analyses were performed by combining a low-Reynolds number k–*ω* model of [14] for the airflow and a Lagrangian stochastic trajectory method for the particulate phase. One of the major findings was that the laminar to turbulent transition was sensitive to the geometry of the airway model, especially for lower flow rates, stressing the importance of considering subject-specific geometries. It was shown that the major percentage of total deposition of particles occurred within the mouth cavity. The flow structures inside an idealized human upper airway were analysed in [15]. The lattice Boltzmann method (LBM) was applied to perform a direct numerical simulation study of the airflow patterns, which were compared with the X-hot-wire anemometry experiments of Johnstone *et al*. [16]. It was demonstrated that the LBM was in good agreement with experimentally observed flow features. The dynamics of airflow in a short inhalation (sniff) for an anatomically realistic geometry (starting from the nose to the second bronchial generation) was computationally studied in [17]. Additionally, the convective transport of a scalar species with relatively low diffusivity (with a Schmidt number of 900) was analysed.

The experimental studies of flow and deposition in models of human airways are necessary for validation of the computer simulations. The local and total deposition measurements in several realistic and throat geometries were performed in [18]. In total, seven representative geometries were studied at flow rates of 30 and $90\;l\;\min^{-1}$, where the range of particles was between 3 and 6.5 μm. The geometry of the models was obtained from the magnetic resonance imaging (MRI) scans. The gamma scintigraphy and gravimetry were used to measure particle depositions. The authors proposed a simple equation correlating the total deposition of particles by introducing the equivalent diameter ($D_{\mathrm{mean}}=2\sqrt{V/\pi L}$, where *V* is the total cast volume and *L* is the length of the central sagittal line of the model) and velocity (*U*_mean_=*QL*/*V* , where *Q* is the volume flow rate) in the Stokes number, which significantly reduced scattering of the data. A steady inspiratory flow, in an anatomically representative model of the human airways, was experimentally investigated in [19]. Magnetic resonance velocimetry (MRV) was used to measure three components of the mean velocity, where water was used as a working fluid. The subject-specific geometry was manufactured from the CT scan images by three-dimensional (3D) printing (stereolithography). It was concluded that both streamwise (due to streamwise gradients) and lateral dispersion (due to secondary flows) were relevant transport mechanisms. This investigation also stressed the importance of considering the subject- and patient-specific geometries instead of simplified airway configurations. In their follow-up study, time-varying flow conditions, which correspond to breathing regimes during moderate exercises, were also analysed with the same technique [20].

In the present study, in addition to a passive distribution of inhaled aerosols, we would like to analyse the potential of a controlled drug-aerosol delivery in the anatomically realistic human respiratory system, based on the magnetic drug targeting (MDT) concept. Magnetic drug delivery involves a straightforward concept of imposing external magnetic field gradients in the proximity of the disease location to act upon the magnetically responsive carriers. An active pharmaceutical ingredient is attached to these magnetic carriers, making it possible to deliver required therapeutic concentrations at pre-specified locations.

Some pre-clinical and phase I/II clinical *in vivo* trials of the MDT technique were reported for treatments of advanced solid malignant tumours [21–23], liver cancer [24,25], prostate cancer [26] and breast cancer [27,28]. Despite these significant advancements of applying the MDT technique, there are still numerous challenges that need to be solved before extensive use of magnetic drug delivery [29–31]. The primary challenge includes a limited exposure of deep organs to sufficiently high magnetic fields to enable an efficient capture of medical drug particles. In our previous work, we demonstrated how computer simulations could contribute to further advancement and optimization of the MDT technique in dealing with localized treatments of cardiovascular diseases [32,33] and brain tumours [34]. In the present contribution, we extend further applications of the MDT technique for potential treatment of respiratory diseases, specifically for localized treatment of a lung cancer.

The motivation of the present research is triggered by the experimental study of the targeted delivery of magnetic aerosol droplets to intact mice lungs, as reported in [35]. It was demonstrated that steering magnetic droplets towards the right lung could be achieved by imposing sufficiently high magnetic field gradients close to the main trachea bifurcation. This magnetic steering enabled an overall fourfold increase of medical drug deposition compared to the neutral situation. In conclusion, Dames *et al*. [35] suggested further developments and scaling-up of the magnetic steering approach to the human lungs. The primary goal of the present research is to check, by performing a series of numerical simulations, to what extent (if at all) it is possible to achieve a magnetic control of drug aerosol distribution in the human airways.

In this work, we focus on computer simulations of the MDT technique to steer and capture magnetic aerosols in an anatomically realistic geometry of human upper and central airways (that extends from the mouth inlet to the eighth generation of bronchial bifurcations) for which a complete 3D velocity field was measured by the MRV technique [19,20]. First, we performed computer simulations of the airflow inside airways to compare the mean flow features with the MRV measurements. Here, the major challenge lies in the local generation of turbulence caused by the formation of the wall jet in the larynx region. Then we analysed the distribution of various classes of the neutral aerosols inside the different parts of the airway system. Finally, aerosols with magnetic core were introduced and a few scenarios of steering and deposition by imposed external magnetic field gradients were analysed.

## 2. Mathematical model

In this section, we provide a comprehensive set of equations used to describe the motion of air and the dynamics of the particulate phase. The airflow dynamics equations are based on the Eulerian approach, whereas the dynamics of the particulate phase are represented in the Lagrangian frame of reference.

### 2.1. Airflow

For the airflow, conservation of mass and momentum in a Cartesian coordinate frame of reference were used:

2.1 $$\frac{\partial U_{i}}{\partial x_{i}}=0$$

and

2.2 $$\frac{\partial U_{i}}{\partial t}+\frac{\partial (U_{i}U_{j})}{\partial x_{j}}=-\frac{1}{\rho }\frac{\partial P}{\partial x_{i}}+\frac{\partial }{\partial x_{j}}\left[(\nu +\nu_{t})\left(\frac{\partial U_{i}}{\partial x_{j}}+\frac{\partial U_{j}}{\partial x_{i}}\right)\right],$$

where *ρ* is the density of fluid and *ν* its kinematic viscosity. As we are dealing with a flow where a local generation of turbulence can occur, we have to account for the turbulence contributions through the turbulent viscosity (*ν*_*t*_). In the present study, we apply two different approaches to model turbulence. The first is based on the Reynolds-averaged Navier–Stokes (RANS) approach, where the turbulent viscosity is evaluated from additional turbulence parameters. For the RANS-SST (shear stress transport) model of [36] adopted here, the following set of four transport equations is used, i.e. the intermittency (*γ*), the transition momentum thickness Reynolds number (*Re*_*θt*_), the TKE (*k*) and, finally, the specific dissipation rate (*ω*). The second simulation approach is the large eddy simulation (LES), which requires that the simulation is run in a time-dependent manner, despite the steady inhalation conditions. Here, the numerical resolving of the flow dominates over its modelled part, which is contained only to flow and turbulence structures smaller than the defined filter size (a size of control volume). Here, we adopted the wall-adopting local eddy-viscosity (WALE) subgrid model of [37] to calculate the subgrid contributions.

#### 2.1.1. Reynolds-averaged Navier–Stokes: SST-transition turbulence model

The transport equation for the intermittency is formulated as follows:

2.3 $$\frac{\partial \gamma }{\partial t}+\frac{\partial (\gamma U_{i})}{\partial x_{i}}=\frac{\partial }{\partial x_{i}}\left[\left(\nu +\frac{\nu_{t}}{\sigma_{\gamma }}\right)\frac{\partial \gamma }{\partial x_{i}}\right]+P_{\gamma }-E_{\gamma },$$

where the main transition source (*P*_*γ*_) and dissipation (*E*_*γ*_) terms are defined as

2.4 $$P_{\gamma }=c_{a1}F_{\mathrm{length}}S{\left[\gamma F_{\mathrm{onset}}\right]}^{1/2}(1-\gamma )\;\mathrm{and}\;E_{\gamma }=c_{a2}\Omega \gamma F_{\mathrm{turb}}(c_{e2}\gamma -1),$$

where *S* is the strain rate magnitude, *Ω* is the vorticity magnitude, while *F*_length_, *F*_onset_ and *F*_turb_ represent intermittency functions, which are designed to properly capture transition from laminar to turbulent flow regime (for detailed derivations of these functions and their finite forms, see [36]). Finally, the remaining model constants are given as *σ*_*γ*_=1, *c*_*a*1_=2, *c*_*a*2_=0.06, *c*_*e*2_=50. The transport equation for transition momentum thickness Reynolds number (*Re*_*θ*t_) can be written as

2.5 $$\frac{\partial Re_{\theta t}}{\partial t}+\frac{\partial (Re_{\theta t}U_{i})}{\partial x_{i}}=\frac{\partial }{\partial x_{i}}\left[\sigma_{\theta t}(\nu +\nu_{t})\frac{\partial Re_{\theta t}}{\partial x_{i}}\right]+P_{\theta t}.$$

The main source term (*P*_*θ*t_) is defined as

2.6 $$P_{\theta t}=\frac{c_{\theta t}}{t}(Re_{\theta t}^{*}-Re_{\theta t})(1-F_{\theta t}),$$

where the Re*_θt_ is the local value calculated from an empirical correlation, *t*=500*ν*/*U*^2^ is the characteristic local time scale, *F*_*θ*t_ is the blending function (for the full expression see [36]) and, finally, *c*_*θ*t_=0.03 and *σ*_*θ*t_=2.0 are model coefficients. The transport equation of the TKE (*k*) is given as

2.7 $$\frac{\partial k}{\partial t}+\frac{\partial (kU_{i})}{\partial x_{i}}=\frac{\partial }{\partial x_{i}}\left[\left(\nu +\frac{\nu_{t}}{\sigma_{k}}\right)\frac{\partial k}{\partial x_{i}}\right]+\gamma_{\mathrm{eff}}P_{k}-\min(\max(\gamma_{\mathrm{eff}},0.1),1.0)Y_{k}.$$

Here, *P*_*k*_ and *Y*_*k*_ are the production and dissipation terms of the turbulence kinetic energy and *γ*_*eff*_ is the effective intermittency calculated from:

2.8 $$\gamma_{\mathrm{eff}}=\max(\gamma ,\gamma_{\mathrm{sep}}),\;\gamma_{\mathrm{sep}}=\min\left(2\;\max\left[0,\left(\frac{Re_{v}}{3.235\;Re_{\theta c}}\right)-1\right]e^{-{\left(R_{T}/20\right)}^{4}},2\right)F_{\theta t},$$

where *Re*_*v*_=*y*^2^*S*/*ν*, *R*_*T*_=*k*/*ω* and *Re*_*θ*c_ are the local strain-rate Reynolds number, the turbulent Reynolds number and the critical Reynolds number where the intermittency first starts to increase in the boundary layer, respectively. Finally, the transport equation for the specific dissipation rate (*ω*) is given as

2.9 $$\frac{\partial \omega }{\partial t}+\frac{\partial (\omega U_{i})}{\partial x_{i}}=\frac{\partial }{\partial x_{i}}\left[\left(\nu +\frac{\nu_{t}}{\sigma_{\omega }}\right)\frac{\partial \omega }{\partial x_{i}}\right]+P_{\omega }-Y_{\omega }+D_{\omega },$$

with *P*_*ω*_ and *D*_*ω*_ as the production terms and *Y*_*ω*_ as the dissipation term. The system of equations (2.1)–(2.9) is finally closed with the expression for the turbulent viscosity, which is written as

2.10 $$\nu_{t}=\min\left[\frac{k}{\omega };\frac{a_{1}k}{SF_{2}}\right],$$

where *a*_1_=0.31 is the model constant and *F*_2_ is the blending function (for definition see [14]).

#### 2.1.2. Large eddy simulation: Wall-adapting local eddy-viscosity subgrid model

Within the LES framework we applied the WALE subgrid model in [37]. This model is selected because of the fact that, in contrast with the standard Smagorinsky model, it does not require any additional empirical wall-damping functions and provides a proper near-wall behaviour of the subgrid turbulent viscosity in the proximity of the wall.

2.11 $$\nu_{t}^{\mathrm{SGS}}=L_{s}^{2}\frac{{\left(S_{ij}^{d}\;S_{ij}^{d}\right)}^{3/2}}{{\left({\bar{S}}_{ij}{\bar{S}}_{ij}\right)}^{5/2}+{\left(S_{ij}^{d}S_{ij}^{d}\right)}^{5/4}},$$

with

2.12 $$\begin{array}{rl} {\bar{S}}_{ij} & \;=\frac{1}{2}\left(\frac{\partial {\bar{U}}_{i}}{\partial x_{j}}+\frac{\partial {\bar{U}}_{j}}{\partial x_{i}}\right),\\ S_{ij}^{d} & \;=\frac{1}{2}\left[{\left(\frac{\partial {\bar{U}}_{i}}{\partial x_{j}}\right)}^{2}+{\left(\frac{\partial {\bar{U}}_{j}}{\partial x_{i}}\right)}^{2}\right]-\frac{1}{3}\delta_{ij}{\left(\frac{\partial {\bar{U}}_{k}}{\partial x_{k}}\right)}^{2}\\ \text{and}\;L_{s} & \;=\min(\kappa d,C_{w}\Delta ), \end{array}\}$$

where ${\bar{U}}_{i}$ is the resolved velocity, *κ* is the von Kármán constant, *d* is the distance to the nearest wall, *Δ*=*V*^1/3^ is the characteristic local grid scale (grid filter) and, finally, *C*_*w*_=0.325 is the model constant.

### 2.2. Aerosol/particulate phase

The Lagrangian approach is applied for the particle tracking and for deposition of particles. This approach is based on a simple balance of all forces (***F***) acting on the particle [38]:

2.13 $$m_{p}\frac{\partial u_{p}}{\partial t}=\sum F_{i},$$

where *m*_p_ is the particle mass and *t* is the characteristic time. After integrating the particle velocity with respect to time, the particle position is calculated as

2.14 $$\frac{dx}{dt}=u_{p}.$$

We next consider the drag, virtual mass, gravity and magnetization forces. The drag force is expressed as

2.15 $$F_{d}=\frac{m_{p}f}{\tau_{p}}(u-u_{p}),$$

where ***u*** is the velocity of the fluid phase and *f* is the drag factor, which depends on the particle Reynolds number, defined as

2.16 $$Re_{p}=\frac{d_{p}}{\nu }|u-u_{p}|$$

and *τ*_p_ is the particle relaxation time, defined as

2.17 $$\tau_{p}=\frac{C_{c}d_{p}^{2}}{18\nu }.$$

In the present study, we assume that the slip correction factor *C*_*c*_=1. We define the Stokes number as the ratio between the characteristic particle relaxation time to the time of the flow:

2.18 $$St=\frac{\rho_{p}d_{p}^{2}\bar{U}}{18\mu D},$$

where $\bar{U}$ is the characteristic velocity and *D* is a typical length scale of the fluid. The drag coefficient is specified as a function of the *Re*_p_ after [38]:

2.19 $$\begin{array}{rl} f & \;=1\;\mathrm{if}\;Re_{p}\leq 1, \end{array}$$

2.20 $$\begin{array}{rl} f & \;=Re_{p}^{0.354}\;\mathrm{if}\;1<Re_{p}\leq 400 \end{array}$$

2.21 $$\begin{array}{rl} \text{and}\;f & \;=1+0.15\;Re_{p}^{0.687}+0.0175{\left(1+4.25\times 10^{4}\;Re_{p}^{-1.16}\right)}^{-1}\;\mathrm{if}\;400<Re_{p}\leq 3\times 10^{5}. \end{array}$$

The virtual mass (added mass) and gravity forces are evaluated as

2.22 $$F_{\mathrm{vm}}=\frac{V_{p}}{2}\frac{D(u-u_{p})}{Dt}\;\mathrm{and}\;F_{g}=m_{p}g.$$

Finally, the magnetization force can be written as

2.23 $$F_{m}=V_{\mathrm{mp}}\mu_{0}M\nabla H,$$

where *V*_mp_ is the volume of the magnetic core of the particle, *μ*_0_ is the magnetic constant, ***M*** is the magnetization and ***H*** is the auxiliary magnetic field. In the present study, we are dealing with fully magnetically saturated particles for which magnetization reduces to ***M***=*M*_sat_***H***/|***H***|, where *M*_sat_ is the saturation magnetization (a material property).

We also define characteristic non-dimensional parameters to quantify the particle deposition efficiency. The local deposition efficiency, defined as the dimensionless surface concentration of the particles, is calculated as

2.24 $$\xi_{\epsilon }(x)=\frac{N_{\mathrm{wall}}(|x_{p,i}-x|<\epsilon )}{N_{\mathrm{in}}},$$

where *ϵ* is the typical search radius from the centre of the wall control volume (e.g. *ξ*_1 mm_ defines that the search radius is *ϵ*=1 mm), *N*_in_ is the number of particles injected at the inlet and *N*_wall_ is the number of the particles captured within the pre-specified search radius (*ϵ*) [34].

## 3. Numerical method

For the gaseous phase (air), the system of equations (2.1)–(2.12) is numerically solved by using the finite-volume approach for arbitrary complex geometries, Ansys/Fluent Inc. 14.0. All diffusive terms in transport equations are discretized by the second-order central differencing scheme (CDS). For the RANS-SST model, the second-order upwinding differencing scheme is used for convective terms in all transport equations. By contrast, the second-order CDS is used in the LES approach for convective terms. Besides, the fully implicit second-order time integration scheme based on the three consecutive time steps is applied in LES. The SIMPLE algorithm is used for predictor/corrector coupling between the velocity and pressure fields. For the particulate phase, equations (2.13) and (2.14) are integrated by the fourth order Runge–Kutta time integration method. The separate user-defined functions are developed which include calculations of the spatial distribution of the auxiliary magnetic field and resulting magnetization force. The former is based on the integration of Biot–Savart’s Law for a current-carrying wire (e.g. [39,40]).

### 3.1. The geometry of the upper and central airways and the numerical mesh used

**Figure 1.**
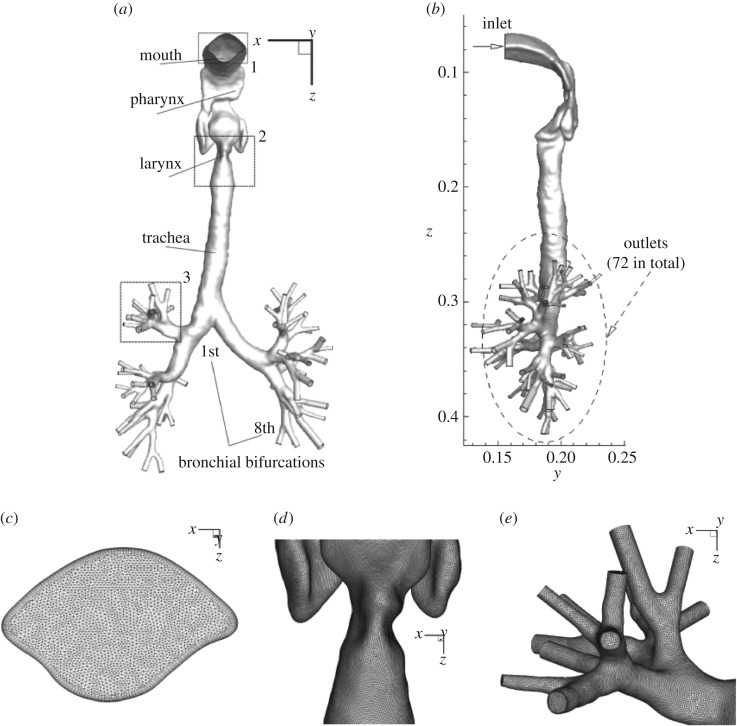
The front view of the geometry of the patient-specific human airway system that extends from the mouth inlet to the eighth generations of the bronchial bifurcations [11,19,20] (*a*); details of the numerical mesh used (zoom-ins)(*a*); a side view of geometry with imposed boundary conditions: a single inlet and multiple outlets (in total 72), where a zero gauge pressure condition is imposed (*b*); details of the numerical mesh (zoom-ins) at 1-2-3 locations (*c*), (*d*) and (*e*), respectively, as indicated in figure 1*a*.

The geometry used is, apart from the inlet region, identical to that of [11] and which was also experimentally studied in [19,20], figure 1*a*,*b*. The CAD geometry was reconstructed from the CT scans with a resolution of 1.25 mm, which was received from [19]. The geometry starts with a part of the mouth and ends with the eighth bronchial generations in the lungs. An Octree-type mesh was selected. The surface mesh was generated first. Then, this smoothed surface mesh was extruded inwards by placing between five (coarse mesh) and 10 (fine mesh) segments to create the prismatic boundary layer. This local mesh refinement is used to more accurately handle possible flow separation and deposition of particles in the near-wall regions. The remaining part of the geometry was then filled using the Delaunay meshing method. Some details of the finest mesh containing approximately 11×10^6^ control volumes and 10 prismatic segments within the boundary layers are shown in figure 1*c*–*e*. All results shown are for the finer mesh.

### 3.2. Imposed boundary conditions

The imposed boundary conditions are also given in figure 1*b*. The walls are treated as the no-slip type of boundary condition. The inlet velocity profiles were assumed to be uniform with the velocity magnitude obtained from the pre-defined value of the Reynolds number, i.e. *V*_0_=(*Re*⋅*ν*)/*D*, where *ν* is the kinematic viscosity of fluid and *D* is the characteristic hydraulic diameter of the airway. The Reynolds number is calculated from the characteristic volumetric flow rate (*Q*) and a characteristic cross-sectional area (*A*) as *Re*=(*Q*⋅*D*)/(*ν*⋅*A*). For a typical flow rate of $Q=60\;l\;\min^{-1}$ of air (or its water equivalent of $3.78\;l\;\min^{-1}$, as used in MRV experiments), which corresponds to deep inspiration when inhaling aerosolized drugs, the inlet Reynolds number is *Re*=3470 (with characteristic inlet of *D*=0.0203 m [19]). For the RANS-SST model, the inlet turbulence intensity is assumed to be 5% and that the ratio between the turbulent and molecular viscosity is *ν*_*t*_/*ν*=10. The zero gauge pressure boundary condition was applied to all outlets (here 72 outlets were imposed in total).

### 3.3. On the adequacy of the numerical mesh used

**Figure 2.**
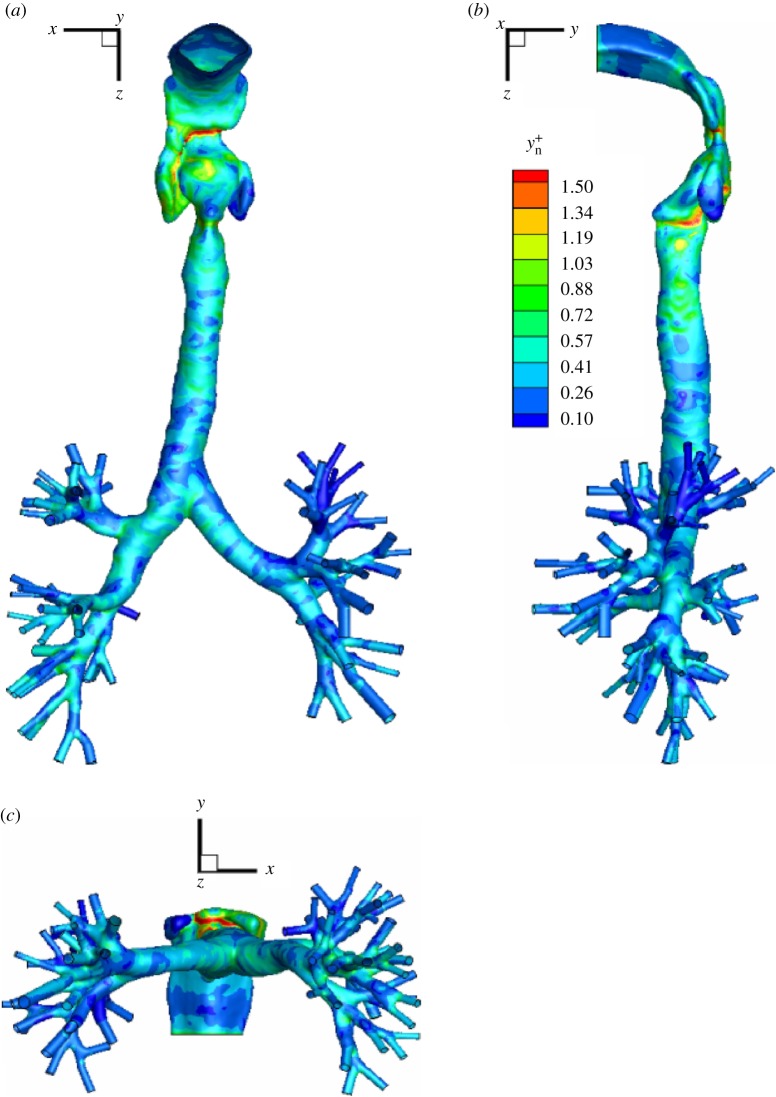
(*a–c*) Contours of the non-dimensional wall distance ($y_{n}^{+}$) in characteristic projections (*x*–*z*, *y*–*z* and *x*–*y* planes). The results are obtained from the low-*Re* RANS-SST simulation.

Because of the expected turbulent character of the flow within the geometry and specified inspiration rate considered, it is important to check to what extent the generated numerical mesh is adequate for the low-Reynolds turbulence closure (within the RANS framework) or for the even more demanding (regarding the spatial mesh resolution) LES approach. The first criterion is evaluated from the low-*Re* RANS-SST results by plotting the non-dimensional wall distance calculated as *y*^+^=*u*_*τ*_⋅*y*_*n*_/*ν*, with the friction velocity $u_{\tau }=\sqrt{\tau_{\mathrm{wall}}/\rho }$ and *y*_*n*_ as the wall-normal coordinate. The wall-shear stress (WSS) is calculated as *τ*_wall_=|*μ*(∂*u*/∂*y*)_wall_|. The contours of the non-dimensional wall distance (*y*^+^_*n*_) are shown in figure 2*a*–*c*. It can be seen that the maximum value of *y*^+^_*n*_≈2 can be observed at particular locations, whereas the criterion that *y*^+^_*n*_≤1 holds for a large part of the simulated domain. This confirms that the numerical mesh is sufficiently refined in the near-wall region. Similarly, in our preliminary study [33], we also estimated the ratio between mesh-based and Kolmogorov length scales ($l_{\mathrm{ratio}}=V_{\mathrm{mesh}}^{1/3}/\eta_{K}$). Here, the *V*_mesh_ is the volume of the characteristic mesh element, whereas the Kolmogorov length scale is evaluated as *η*_*K*_=(*ν*^3^/*ε*)^1/4^ (*ε* from the low-*Re* RANS-SST is used). For a well-resolved LES, it is to be specified that this ratio is between 5 and 10. We found that apart from a relatively narrow range of 0.13≤*z*≤0.15 *m*, this condition was also satisfied. It can be concluded that the carefully designed locally refined numerical mesh with prismatic boundary layers is appropriate for the well-resolved LES.

## 4. Results and discussion

### 4.1. Mean flow features: comparative assessment of Reynolds-averaged Navier–Stokes, large eddy simulation and experiments

We focus first on the comparative assessment of the velocity field obtained by numerical simulations (RANS and LES) and MRV experiments by Banko *et al*. [19]. Note that the RANS-SST simulations are performed in the steady mode, whereas the wall-resolved LES are performed in the time-dependent mode. The characteristic value of the time step is Δ*t*=10^−4^ *s*, which ensures that the CFL (Courant–Friedrichs–Lewy) number is less than one. For LES, after five initial flow-through times, collecting of first- and second-order statistics is started and continued over a period of an additional 20 flow-through times.

**Figure 3.**
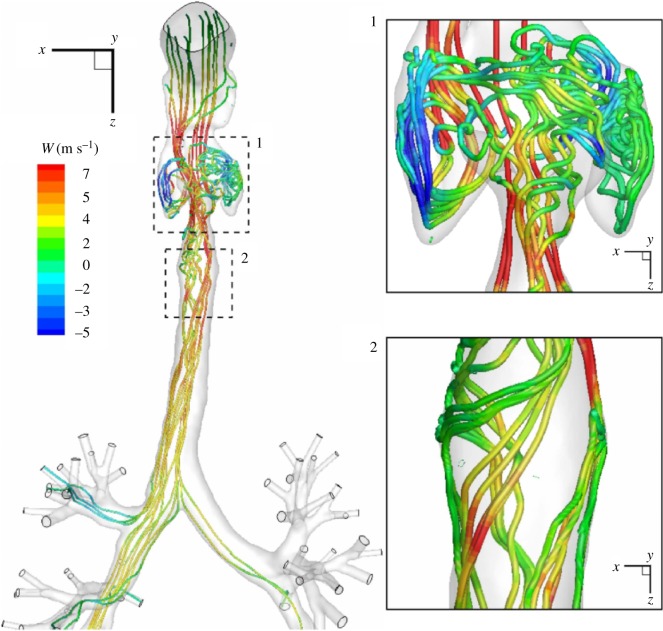
A front view of the streamtraces coloured with the instantaneous velocity component in the *z*-direction (*W* in m s^−1^) for an arbitrary time instant—results obtained with the LES-WALE (left). The zoom-ins in characteristic regions of interest: 1—oesophageal space and glottis; 2—trachea.

**Figure 4.**
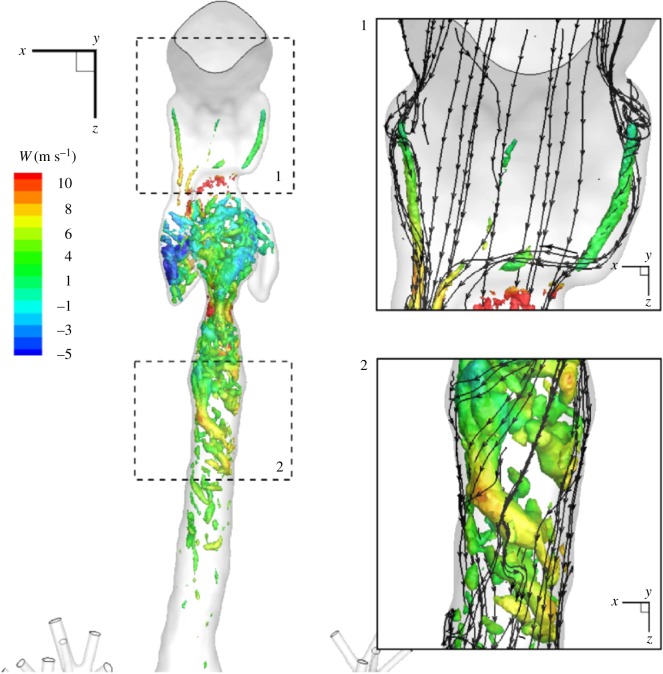
A front view of the isosurface of the pressure Laplacian (∇^2^*p*=10^6^ (in Pa m^−2^)) coloured with the velocity component in the *z*-direction (*W* in m s^−1^) (left). The zoom-ins in characteristic regions of interest: 1—oropharynx and 2—trachea, with characteristic streamtraces.

To portray the most salient features of the airflow, 3D streamtraces coloured with the streamwise velocity are plotted in figure 3. Note that for these plots we used the instantaneous velocity field obtained from the LES-WALE simulation at an arbitrary time instant. It can be seen that the flow recirculation (identified by the negative *W*-velocity component) is generated within the oesophageal space (the left and right extensions), while a strong jet motion is generated in the epiglottis (the upper/first narrowing) and glottis (the lower/second narrowing) cross sections; figure 3, zoom-in (1). After entering the trachea, the flow pattern is characterized by a strong swirling behaviour, as seen in figure 3, zoom-in (2). To identify more quantitatively the swirling eddy structures (vortex cores), we calculated the Laplacian of the pressure field (∇^2^*p*) in the entire domain, and plotted the selected isosurface coloured by the velocity component in the *z*-direction (*W*); figure 4. Again, it is observed that the densest region extends from the very first narrowing (the epiglottis) to the first half of the trachea. The first swirling structures are generated along the side walls of the oropharynx; figure 4, zoom-in (1). A similar zoom within the trachea indicates a strong swirling flow that gradually decays towards the first bifurcation; figure 4, zoom-in (2). Note that, in contrast with streamtraces, plotting of the vortical structures gives a potential indication where the turbulence can be present.

**Figure 5.**
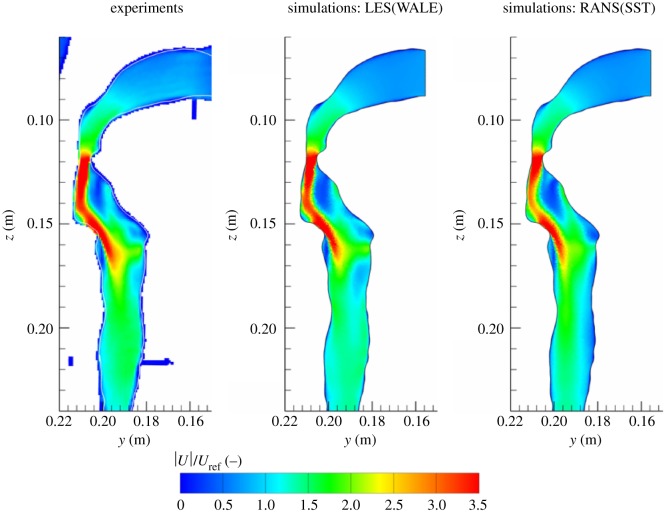
Contours of the non-dimensional modulus of velocity (|*U*|/*U*_ref_) in the central vertical cross section of the geometry shown in figure 1: experiments (MRV of Banko *et al*. [19]), LES with the WALE subgrid model and RANS with the SST model. The *U*_ref_ is the characteristic velocity in the trachea (as specified in [19], *U*_ref_=0.22 m s^−1^ for a water model flow). In the present case, *U*_ref_=3.5 m s^−1^ for the airflow.

The contours of the non-dimensional modulus of velocity (|*U*|/*U*_ref_), in the central vertical cross section, are shown in figure 5. Because of the limited spatial resolution of measurements, for experimental data, we have also included the wall boundaries (as white lines in all contour plots) from the geometry (STL) file. Simulations properly captured the location where the formation of the laryngeal jet takes place as well as its overall shape. Compared to experiment, the RANS-SST simulation slightly underpredicts the strength of the jet in 0.135≤*z*≤0.145 m region. Consequently, the region downstream from the laryngeal jet also shows some differences. It can be concluded that good agreement between simulations and experiment is obtained concerning predictions of the laryngeal jet behaviour.

**Figure 6.**
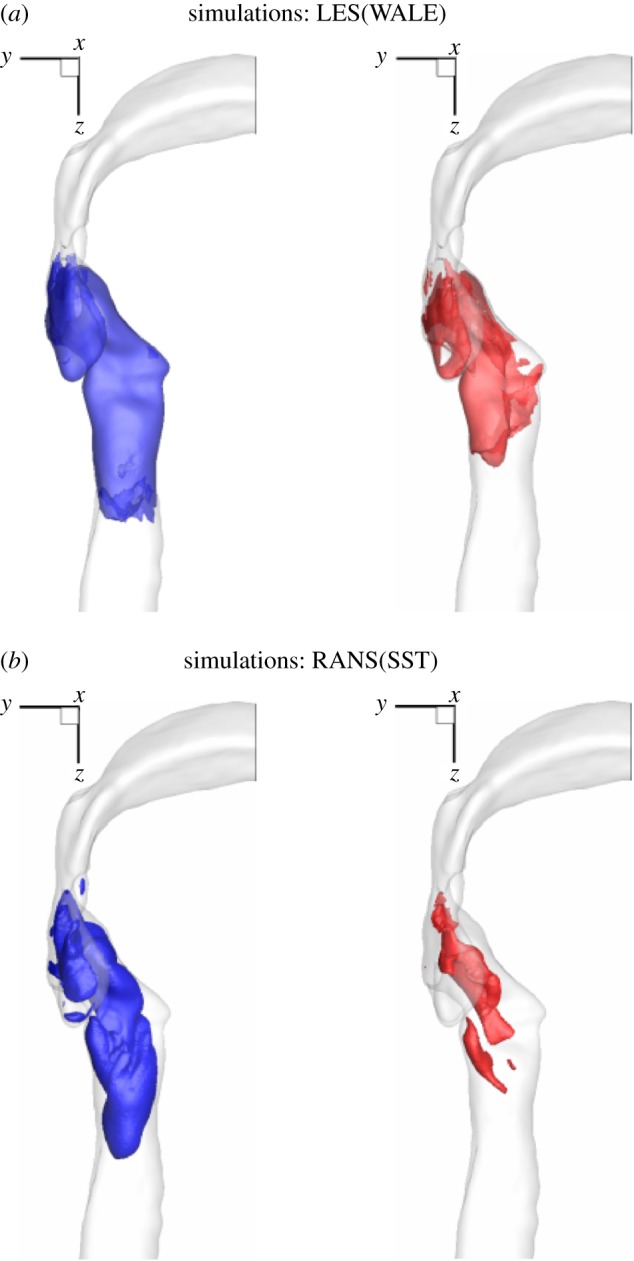
Isosurfaces of the turbulent kinetic energy (TKE) ($k=0.5(\bar{uu}+\bar{vv}+\bar{ww})$: blue 2 m^2^ *s*^−2^, red 4.5 m^2^ *s*^−2^) obtained with the LES (*a*) and RANS with the SST model (*b*).

Two selected isosurfaces of the turbulent kinetic energy (TKE) are shown in figure 6. Note that experimental data do not provide this quantity, so only simulations results are compared. The distribution of the TKE nicely portrays the local generation of turbulence within the laryngeal jet and its relatively rapid decay in the downstream region. This distribution corresponds to regions where the most dense population of vortical structures is observed, as shown in figure 4. It can be seen that both simulations capture similar regions. The LES results (figure 6*a*) indicate stronger contributions of the velocity fluctuations compared to the RANS (figure 6*b*) results, but the overall agreement is good.

**Figure 7.**
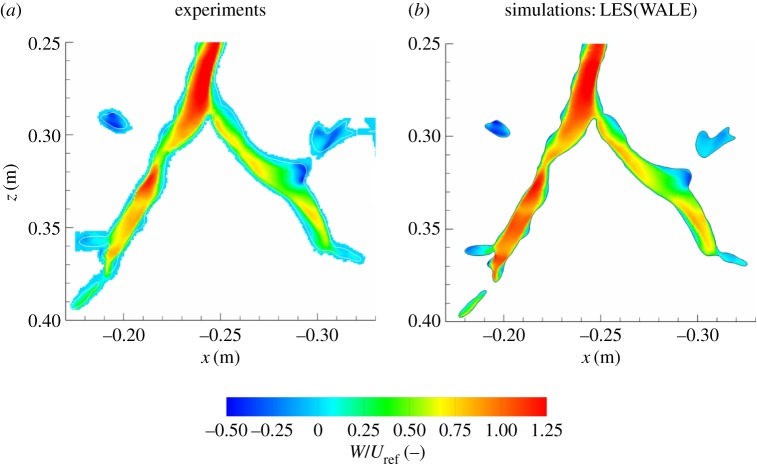
Contours of the non-dimensional vertical velocity (*W*/*U*_ref_) in the central vertical cross section of the geometry shown in figure 1: (*a*) experiment (MRV of [19]), (*b*) LES with the WALE subgrid model.

Next, we move closer to the regions just after the first bifurcation; figure 7. Here, we compare measured and simulated (LES) non-dimensional *z*-velocity components. The velocity contours show good agreement for the incoming jet in the 0.25≤*z*≤0.3 m region. Then, flow splits asymmetrically with a distinctly higher intensity of velocity in the left branch. In the left branch, the LES shows slightly higher velocity compared to measurements in the *z*>0.34 m region. In the right branch after bifurcation, the flow behaviour is well captured. This includes the recirculation zone in the 0.32≤*z*≤0.33 m, as well as the jet deflection towards the left/lower wall within the right branch.

**Figure 8.**
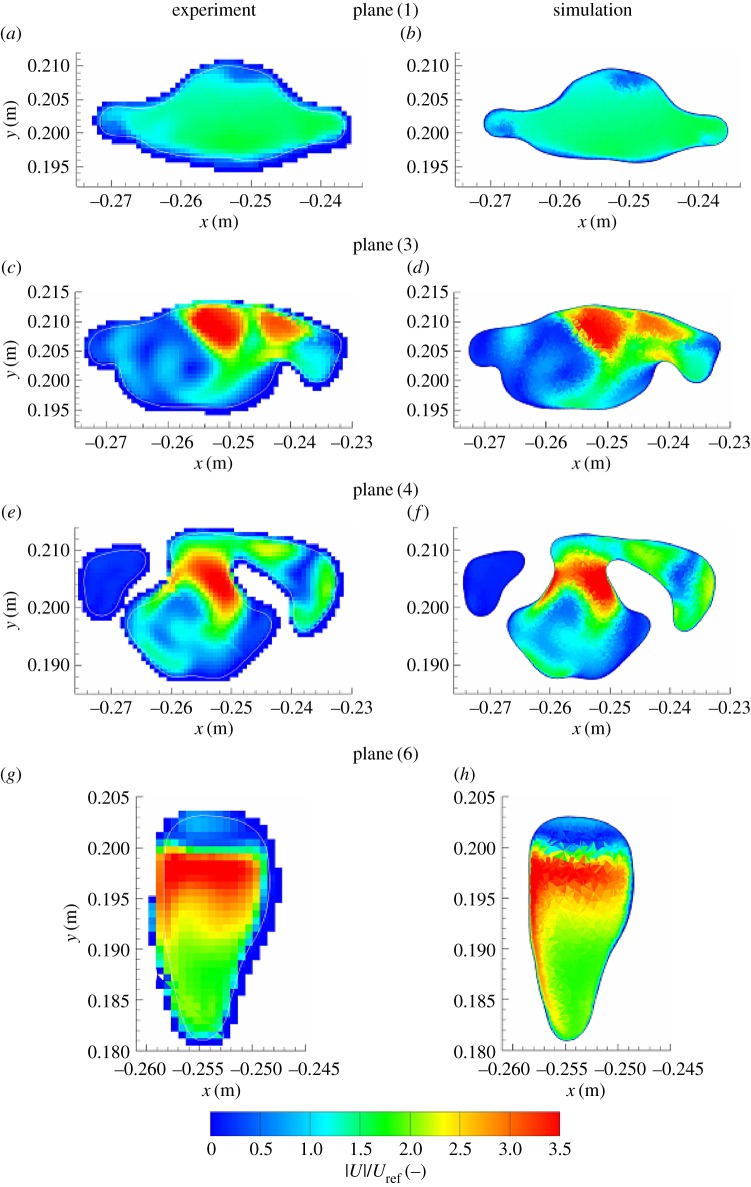
(*a–h*) Contours of the non-dimensional modulus of velocity (|*U*|/*U*_ref_) at various horizontal cross sections (plane(1)–plane(3)–plane(4)–plane(6) at *z*=0.1, 0.135, 0.146 and 0.161 m, which are also indicated in figures 10*a*, 11*a*, 12*a* and 13*a*, respectively) of the geometry shown in figure 1: left—experiments (MRV of [19]), right—LES with the WALE subgrid model.

**Figure 9.**
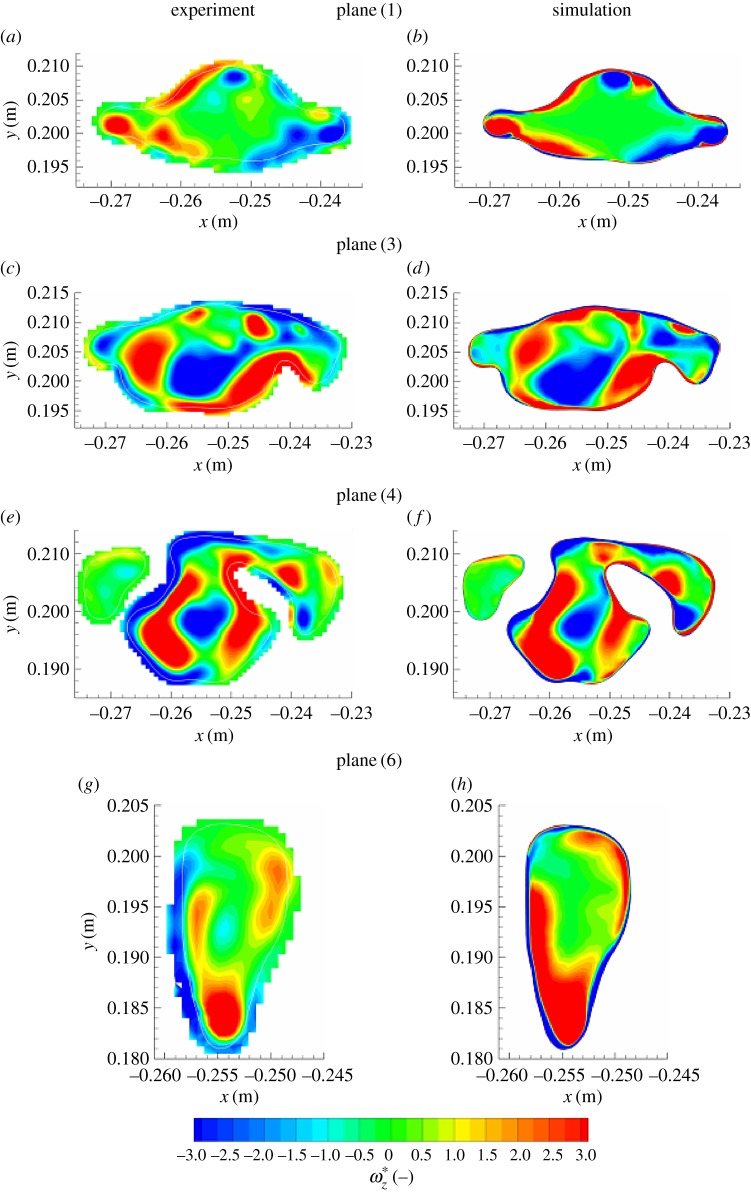
(*a–h*) Same as in the previous figure, only now for the non-dimensional vertical vorticity ($\omega_{z}^{*}=\omega_{z}D/U_{\mathrm{ref}}$).

We next analyse distributions of the velocity and vorticity in selected horizontal cross sections (i.e. in the *x*–*y* planes perpendicular to the main flow direction), figures 8 and 9. Here, we focus mainly on the pharynx and post-laryngeal regions (figure 1*a*,*b*), where the flow exhibits the most complex behaviour, which includes helical motion (swirling), separation, strong acceleration and recirculation. The four pre-selected planes (plane (1), plane (3), plane (4) and plane (6)) are defined with *z*=0.1, 0.135, 0.146 and 0.161 m (figure 5), respectively. It can be seen that a good agreement between the experiment and simulations (LES) is obtained at practically all locations. All experimentally captured (figure 8*a*,*c*,*e*,*g*) salient flow features are also visible in simulations (figure 8*b*,*d*,*f*,*h*). There is just a slight disagreement in plane (6), where the simulation shows a somewhat more extended left-side-oriented jet-like structure compared to the experiment. The contours of the non-dimensional vertical vorticity ($\omega_{z}^{*}=\omega_{z}\cdot D/U_{\mathrm{ref}}$, *ω*_*z*_=−(∂*u*_*x*_/∂*y*−∂*u*_*y*_/∂*x*)) in identical planes (plane (1)–plane (6)) are shown in figure 9. Note that the vorticity is a more sensitive parameter relative to the velocity magnitude because it contains derivatives of both velocity components in the particular plane. It can be seen that overall behaviour is relatively well captured in simulations, but there are some minor differences at specific locations. Differences in the proximity of walls can be explained by the lower spatial resolution of the measurements, which is particularly important in the near-wall regions.

**Figure 10.**
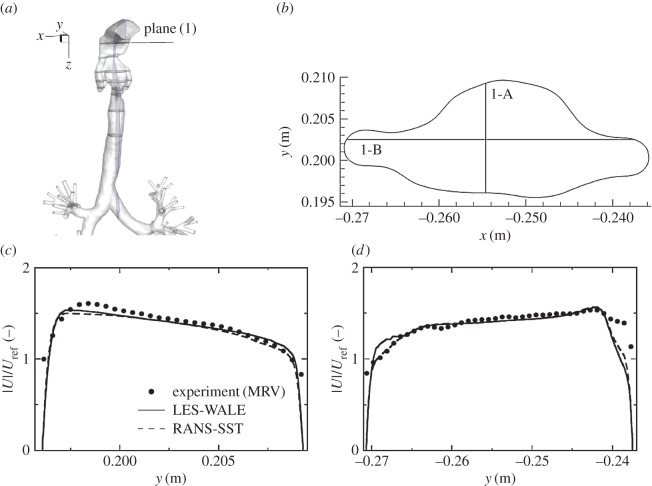
Comparison of the characteristic velocity magnitude profiles (MRV experiment, RANS-SST, and LES) extracted along the vertical and horizontal lines (1-A and 1-B), (*c*) and (*d*), respectively, depicted in (*b*) at specified horizontal cross section, plane (1) (*a*).

**Figure 11.**
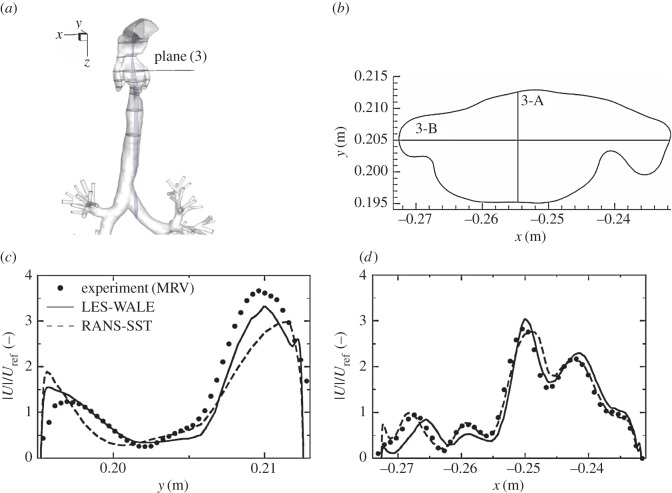
Same as in figure 10, only for the cross section in plane (3).

**Figure 12.**
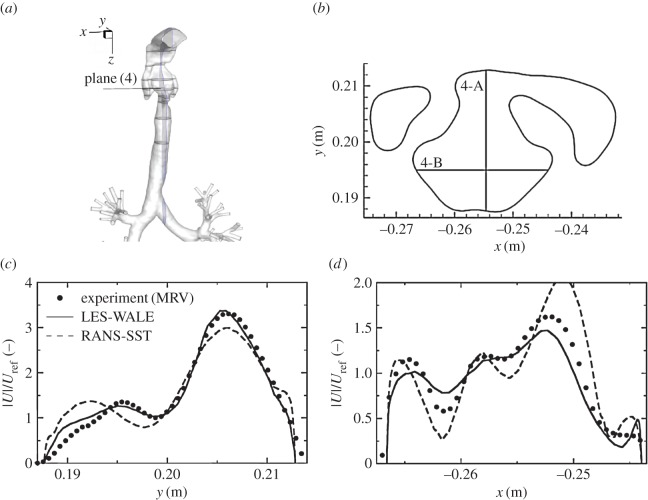
Same as in figure 11, only for the cross section in plane (4).

**Figure 13.**
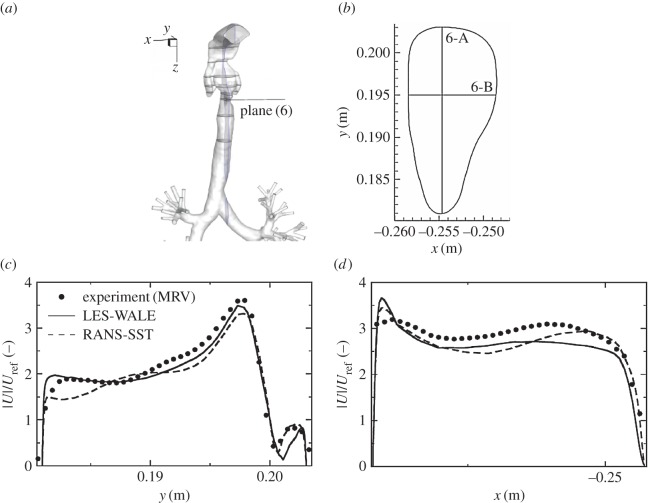
Same as in figure 12, only for the cross section in plane (6).

To provide an even more detailed comparison, we extract profiles of the velocity magnitude in particular horizontal planes along pre-specified lines (A and B) perpendicular to each other, as shown in figures 10–13. In plane (1), the velocity profiles exhibit a simple monotonic behaviour portraying relatively uniform distributions in the central part of the domain, figure 10*c*,*d*. A very good agreement is obtained except for a few points located in the proximity of walls, but this can be explained in terms of lower experimental resolution and overly blurred pixels here, which is again reflected by showing some non-physical values within the wall. Note also that there is practically no difference between RANS-SST and LES results, which can be explained in terms of the relatively low intensity of turbulence at this location. Already at the location of plane (3), the velocity profiles exhibit more complex multiple-peak distributions; figure 11. Along the 3-A line, the LES result shows slightly better predictions of the peaks in the proximity of walls compared to RANS-SST. But again, a good agreement between LES, RANS-SST and experiments is obtained. For the 4-A and 4-B locations, the RANS result slightly overpredicts the experimental and LES peaks, but peak locations are well predicted; figure 12*c*,*d*. Finally, profiles in plane (6) are shown in figure 13. Along the 6-A line, the RANS-SST slightly underpredicts the peak values. In contrast to that underprediction, along the 6-B line the RANS-SST captures the second peak in good agreement with experiments.

Finally, based on all the presented comparisons between the water model experiment and simulations (figures 5–13), it is concluded that generally a good agreement is obtained at all locations considered. The LES results proved to be in a slightly better agreement with experiment than the RANS-SST. Despite this, considering the total computation costs between the LES and RANS-SST used here (the LES are at least $O(10^{2})$ more computationally expensive due to a necessity to perform the time-dependent simulations even for the steady inspiration conditions) and that the RANS-SST model properly captured practically all of the most salient flow features in the upper human airways, we decided to use the RANS-SST model in the context of the multiphase simulations that will include Lagrangian tracking of injected aerosols.

### 4.2. Multiphase simulations of aerosol transport and deposition

**Figure 14.**
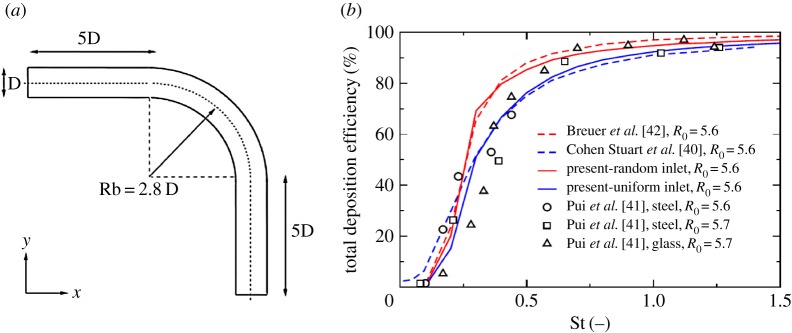
Benchmark solutions for the 90° bend geometry validated against numerical simulations of [42,40] and experiments of [41]. *Re*_*D*_=1000, *R*_0_=*R*_*b*_/*R*_tube_= 5.6, *St*=0.1−1.5.

**Figure 15.**
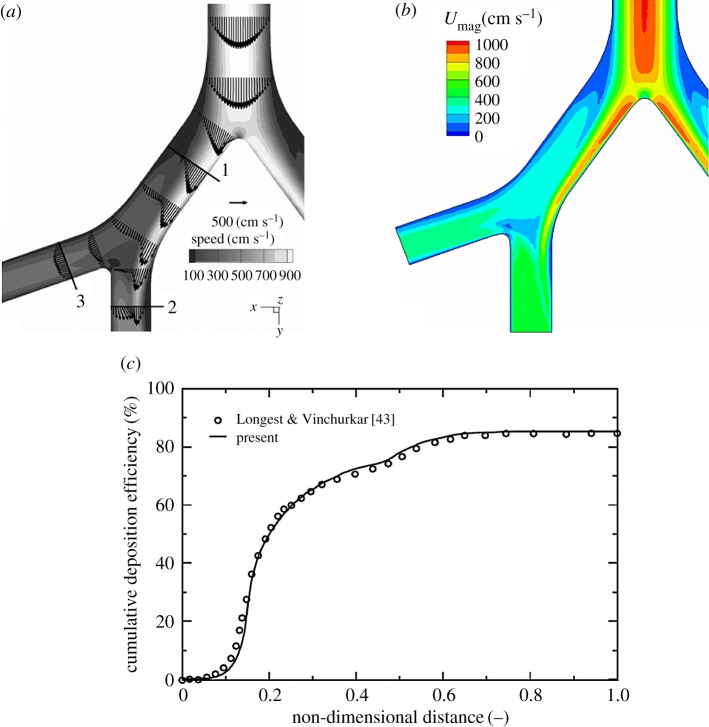
Benchmark solution for the double bifurcation geometry of [43] with fully developed laminar inlet conditions at *Re*=1788: (*a*) the referent velocity vectors and magnitude from [43]; (*b*) contours of the velocity magnitude from the present study; (*c*) comparison of the cumulative deposition efficiency for *St*=0.25 particles. The distance between outlet and inlet is used for non-dimensional distance.

Prior to full multiphase simulations of aerosol distribution in the human upper and central respiratory system, we performed a series of simulations in simplified geometries for which experimental and numerical results of other authors are available. The first configuration is a 90° bent tube with characteristic curvature ratio (*R*_0_=*R*_bent_/*R*_tube_=5.6 giving a Dean number of *De*=423) and with a fully developed laminar inflow (*Re*_*D*_=1000) with non-magnetic particles, previously experimentally studied in [41] and numerically in [42,40]. Here, we study effects of the generated secondary motion on the particle distributions. The sketch of the geometry under study as well as specification of different classes of particles (with a particle diameter range 20≤*d*_p_≤85 μm) are shown in figure 14. It is shown that the total deposition efficiency agrees very well with experiments and with other numerical studies for all simulated classes of particles, confirming the accuracy of the present approach. In the second simplified geometry case, we address flow and particle deposition in a double bifurcation geometry, identical to [43]. Despite simplifications, this geometry contains many flow features occurring in real human airways. Here, the air is a working fluid and particles have a diameter of *d*_p_=10 μm and a density of *ρ*_p_=1060 kg m^−3^. It can be seen that both velocity distributions and cumulative deposition efficiency agree very well with results of [43] (figure 15). We conclude that the present results are in good agreement with the two above-mentioned benchmark studies in predicting both integral and cumulative deposition efficiencies, and as a next step, we simulate the geometry of the human respiratory system (as shown in figure 1). It is noted that here we combine the RANS-SST model for the airflow and the Lagrangian tracking for the particulate phase.

The particles are uniformly released at the inlet plane. As a very diluted concentration of the aerosols is inhaled, we assume one-way coupling between particles dynamics and airflow. Furthermore, the particle–particle interactions are neglected. This makes it possible to simultaneously simulate various classes of particles in a single computational run. The particle–wall interactions are assumed to be fully inelastic. This is due to the presence of the adhesive mucous layer surrounding the airway walls. To obtain reliable statistics of both local and total deposition efficiencies, in total 3×10^5^ particles per particle diameter class (0.1 μm≤*d*_p_≤10 μm) are injected at the inlet.

**Figure 16.**
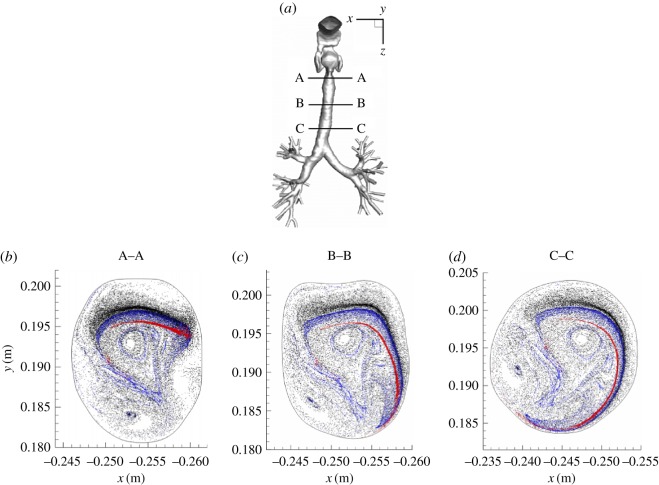
Particle distributions in characteristic cross sections (A–A, B–B and C–C) along the trachea. Black *d*_p_=0.1 μm; blue *d*_p_=3 μm; red *d*_p_=5 μm, respectively.

**Figure 17.**
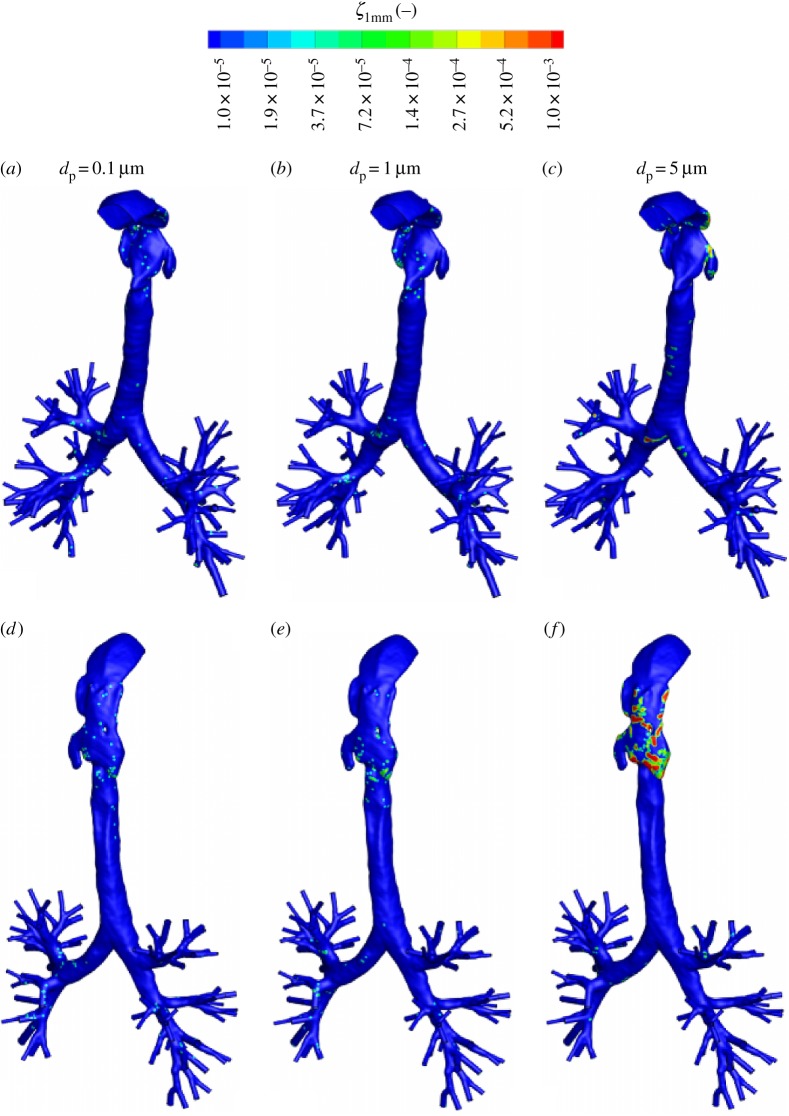
The contours of the local deposition efficiency (*ξ*_1 mm_) for three characteristic classes of non-magnetic particles: *d*_p_=0.1 μm (*a*) and (*d*), 1 μm (*b*) and (*e*), and 5 μm (*c*) and (*f*), respectively. A view from front (*a*)–(*c*) and a view from back (*d*)–(*f*).

The distribution of different classes of the aerosol particles at specific planes (A–A, B–B, C–C) within the trachea are shown in figure 16. These plots are generated by recording locations where the aerosol particles are crossing the specified horizontal planes. It can be seen that for the smallest particles (*d*_p_=0.1 μm) strongest scattering is observed, whereas the larger particles (*d*_p_=3 and 5 μm) are confined within significantly smaller regions. For all classes of particles, a strong asymmetry in distribution is caused by the presence of substantial helical airflow pattern within the trachea. The contours of the local deposition efficiency (*ξ*) with a search radius of *ϵ*=1 mm, for different classes of the neutral (non-magnetic) particles (*d*_p_=0.1,1 and 5 μm), are shown in figure 17. It can be seen that the particles deposit primarily at the back of the pharynx (for all *d*_p_) and at bifurcations (for particles with smaller *d*_p_=0.1 and 1 μm). The local concentration of deposited particles at the back of the pharynx increases with increasing a particle diameter; figure 17*d*–*f*.

**Figure 18.**
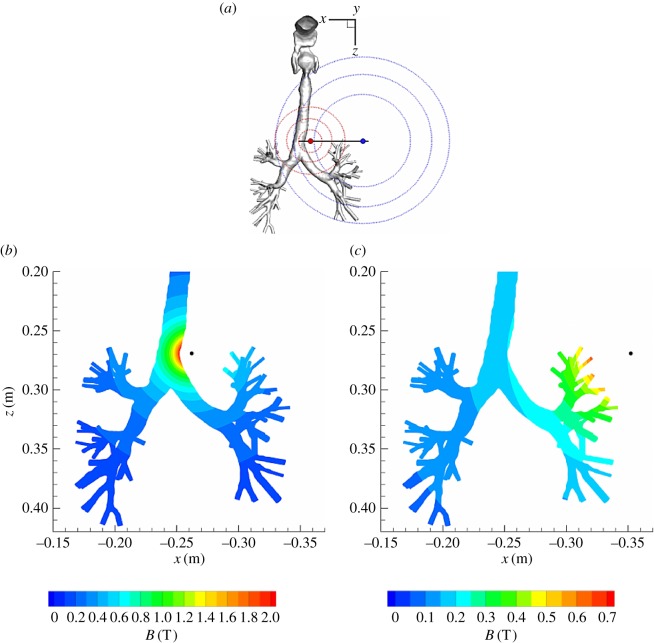
Schematic of the magnetic source (*a*) and contours of the magnetic field intensity for two locations, which are defined as a distance between the trachea wall and magnetic source, *r*_mag_=1 cm (*x*_mag_=−0.252 m, *z*_mag_= 0.269 m) (*b*) and *r*_mag_=10 cm (*x*_mag_=−0.352 m, *z*_mag_=0.269 m) (*c*), respectively.

In the next step, we apply an external magnetic field and investigate its impact on the behaviour of magnetic particles. As a proof of concept study, we consider two situations, which are characterized with different distances from the point-like magnetic source as illustrated in figure 18. The magnetic source is placed at the right side from trachea just above the first bifurcation; figure 18*a*. The characteristic distances between the magnetic source and the wall of the trachea are 1 cm and 10 cm, respectively. The strength of the generated magnetic field (≈2 *T*) corresponds approximately to the values of magnetic field in the present generation of MRI scanners in clinical applications. A colour map of the spatial distribution of the imposed magnetic field for these two distances are plotted in figure 18*b*,*c*, respectively. For the two situations, the strength of the electric current through the point-like (wire) source is the same, resulting in a magnetic field strength along the trachea of 2 T and 0.2 T, for distances of *r*_mag_=1 cm and *r*_mag_=10 cm, respectively.

In the present investigation, we define two classes of magnetic particles. We introduce a characteristic ratio of the magnetic-core (*d*_*m*_) and total (*d*_p_) diameters, defined as $d_{\mathrm{mp}}^{*}=d_{m}/d_{p}$. When $d_{\mathrm{mp}}^{*}=1,$ we have fully magnetic particles, whereas, for $d_{\mathrm{mp}}^{*}=0,$ we have magnetically neutral particles. For the magnetic drug delivery, we introduce particles containing the magnetic core. The shell is a mixture of a medical drug and a carrier. For the carrier, we adopt the biodegradable poly(lactide-co-glycolide), commonly referred to as the PLGA. The resulting density of such a shell particle can be calculated as

4.1 $$\rho_{p}=\rho_{\mathrm{core}}{\left(d_{\mathrm{mp}}^{*}\right)}^{3}+\rho_{\mathrm{shell}}[1-{\left(d_{\mathrm{mp}}^{*}\right)}^{3}].$$

As the shell contains both magnetic carrier and medical drug, its density depends on the medical drug loading. The latter is expressed as a mass percentage of the medical drug in the shell layer. Finally, the shell density is calculated as

4.2 $$\rho_{\mathrm{shell}}={\left(\frac{f_{l}}{\rho_{\mathrm{drug}}}+\frac{1-f_{l}}{\rho_{\mathrm{carrier}}}\right)}^{-1},$$

where *f*_*l*_ is the fractional loading of the medical drug within the shell layer (based on the mass). In the present study, the magnetic core is made from the iron oxide-maghemite (*γ*Fe_2_O_3_), with a density of *ρ*=4860 kg m^−3^ [44], magnetic susceptibility *χ*_*m*_=3 [45] and saturation magnetization *M*_sat_=3.9×10^5^ *A* m^−1^ [46]. The shell carrier is PLGA with a characteristic density of 1300 kg m^−3^. The fractional loading of the medical drug is taken to be *f*_*l*_=30%, which corresponds to an anti-tubercular drug, with a typical density of 1610 kg m^−3^ [47].

**Figure 19.**
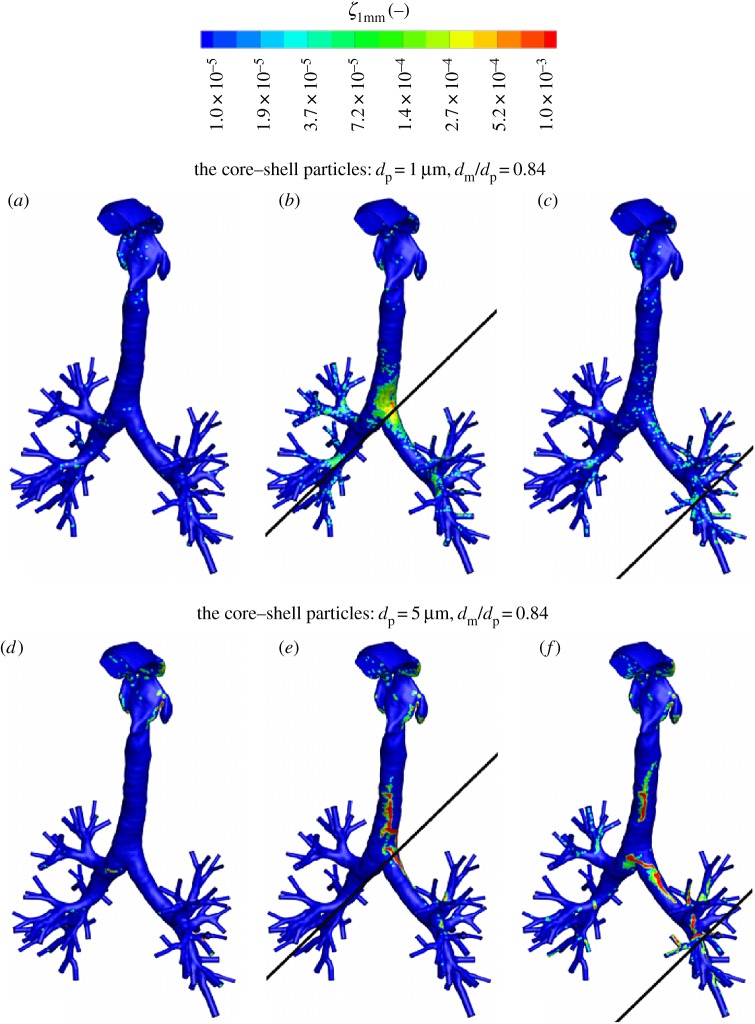
The contours of the local deposition efficiency (*ξ*_1 mm_) for two classes of the magnetic core–shell particles with *d*_p_=1 μm ((*a*)–(*c*)) and 5 μm ((*d*)–(*f*))—both with *d*_*m*_/*d*_p_=0.84. No magnetic field ((*a*) and (*d*)), the magnetic field in case with *r*_mag_=1 cm ((*b*) and (*e*)) and *r*_mag_=10 cm ((*c*) and (*f*)) distance from the magnetic source, as shown in figure 18.

The resulting local deposition efficiency of the shell–core magnetic particles is shown in figure 19. For illustration, we selected the core–shell particles with *d*_p_=1 μm and 5 μm, and with $d_{\mathrm{mp}}^{*}=d_{m}/d_{p}=0.84$. Compared to the neutral situation shown in figure 19*a*,*d*, contours indicate a significant enhancement of the particle deposition efficiency in the proximity of the magnetic sources, as shown in figure 19*b*,*c*,*e*,*f*. With increasing distance between the magnetic source and the targeted regions, from *r*_mag_=1 cm to a more realistic distance of *r*_mag_=10 cm for the entirely non-invasive treatment (figure 19*c*,*f*), the local deposition efficiency is still increased in comparison to the neutral case.

**Figure 20.**
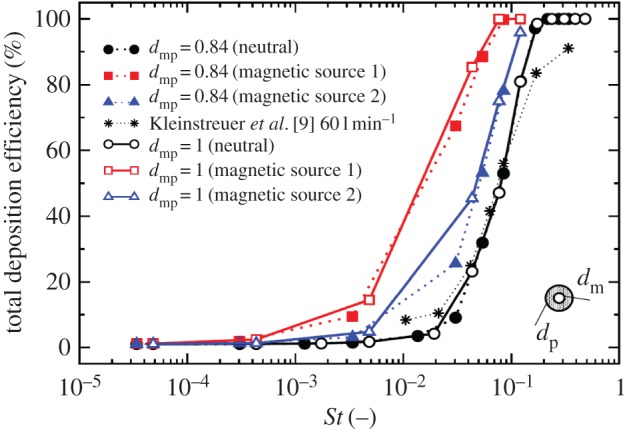
The total deposition efficiency for the different classes of neutral and magnetic particles (a fully magnetic ($d_{\mathrm{mp}}^{*}=1$), and shell–core magnetic particles ($d_{\mathrm{mp}}^{*}=0.84$)) for two characteristic locations of the magnetic point-like sources, as shown in figure 18.

The total deposition efficiency (which is defined as a ratio of the number of the particles deposited along the walls and the number of the injected particles) for the neutral and for two classes of the magnetic particles ($d_{\mathrm{mp}}^{*}=1$ and 0.84, respectively) for the previously analysed two different locations of the magnetic sources, is plotted in figure 20. The experimental results of [9] are also added for a qualitative comparison. No significant effect of the magnetization can be observed in the range *St*≤5×10^−4^. For the *St*>0.1 range, practically all particles found are deposited along the walls. The dependence observed for the non-magnetic particles shows a qualitatively similar behaviour to the results of [9]. The magnetic modulation is the most effective in the 5×10^−4^≤*St*≤10^−1^ range. This range corresponds to particles with a diameter approximately between 0.3 and 5 μm. For example, at *St*=0.05, the total deposition efficiency has increased from 23% for the neutral particles to 46% and 85% for the magnetic particles for the magnetic source distances of *r*=10 cm and 1 cm, respectively. Similar analysis for *St*=0.005 shows that the efficiency increased from a rather low 2% for neutral particles to 5% and 15% for magnetic sources 2 and 1, respectively. It can be noted that relatively small differences are obtained between the fully magnetic (solid lines) and core–shell particles (dashed lines) confirming potentials of the MDT technique to deliver medical drugs coated around a magnetic core. This kind of information, together with maps of the local concentrations of the deposited particles, can be used for improvements of the existing generation of magnetically neutral medical aerosols, as well as for the design of a new generation of medical drugs with the magnetic core.

## 5. Conclusion

We applied numerical simulations to describe the mean airflow patterns and distributions of aerosols in an anatomically realistic geometry of the human airways under the steady inspiration condition with a flow rate of $60\;l\min^{-1}$. The geometry considered extended from the mouth inlet to the eighth bronchial generations. The results obtained with RANS-SST and LES-WALE were compared against MRV experiments under identical conditions. A good agreement between simulations and measurements was obtained at all locations considered. Despite the fact that a slightly better agreement with experiments was obtained with the LES compared to the RANS, the Lagrangian tracking of the particulate phase was performed in conjunction with the RANS-SST model due to significantly lower computational costs. We covered an extensive range of particle sizes, ranging from 0.1 to 10 μm, without and with the magnetic core. For the latter, to mimic as realistically as possible the structure of the pharmaceutical medical drugs, we have selected some of the recently proposed aerosol carriers based on the shell–core concept. We found that the local and total deposition efficiencies can be significantly enhanced by activation of the magnetization force. The most effective enhancement was observed in the 5×10^−4^≤*St*≤10^−1^ range, which corresponds to particles with a diameter approximately between 0.3 and 5 μm.

## Data accessibility

The data supporting the findings of this study are available at the Dryad Digital Repository (http://dx.doi.org/10.5061/dryad.0jt43) [48].
